# Reciprocal regulation of NRF2 by autophagy and ubiquitin–proteasome modulates vascular endothelial injury induced by copper oxide nanoparticles

**DOI:** 10.1186/s12951-022-01486-7

**Published:** 2022-06-11

**Authors:** Na Li, Hang Du, Lejiao Mao, Ge Xu, Mengling Zhang, Yinzhen Fan, Xiaomei Dong, Lijun Zheng, Bin Wang, Xia Qin, Xuejun Jiang, Chengzhi Chen, Zhen Zou, Jun Zhang

**Affiliations:** 1 Molecular Biology Laboratory of Respiratory Disease, Institute of Life Sciences, Chongqing Medical University, Chongqing, 400016 People’s Republic of China; 2 Chongqing Prevention and Treatment Center for Occupational Diseases, Chongqing Key Laboratory of Prevention and Treatment for Occupational Diseases and Poisoning, Chongqing, 400060 People’s Republic of China; 3 College of Pharmacy, Chongqing Medical University, Chongqing, 400016 People’s Republic of China; 4 Department of Pharmacy, The First Affiliated Hospital of Chongqing Medical University, Chongqing, 400016 People’s Republic of China; 5 Center of Experimental Teaching for Public Health, Experimental Teaching and Management Center, Chongqing Medical University, Chongqing, 400016 People’s Republic of China; 6 Department of Occupational and Environmental Health, School of Public Health, Chongqing Medical University, 400016 Chongqing, People’s Republic of China; 7 Research Center for Environment and Human Health, School of Public Health, Chongqing Medical University, Chongqing, 400016 People’s Republic of China

**Keywords:** CuONPs, Vascular injury, NRF2, Autophagy, Ubiquitin–proteasome system

## Abstract

NRF2 is the key antioxidant molecule to maintain redox homeostasis, however the intrinsic mechanisms of NRF2 activation in the context of nanoparticles (NPs) exposure remain unclear. In this study, we revealed that copper oxide NPs (CuONPs) exposure activated NRF2 pathway in vascular endothelial cells. *NRF2* knockout remarkably aggravated oxidative stress, which were remarkably mitigated by ROS scavenger. We also demonstrated that KEAP1 (the negative regulator of NRF2) was not primarily involved in NRF2 activation in that *KEAP1* knockdown did not significantly affect CuONPs-induced NRF2 activation. Notably, we demonstrated that autophagy promoted NRF2 activation as evidenced by that *ATG5* knockout or autophagy inhibitors significantly blocked NRF2 pathway. Mechanically, CuONPs disturbed ubiquitin–proteasome pathway and consequently inhibited the proteasome-dependent degradation of NRF2. However, autophagy deficiency reciprocally promoted proteasome activity, leading to the acceleration of degradation of NRF2 via ubiquitin–proteasome pathway. In addition, the notion that the reciprocal regulation of NRF2 by autophagy and ubiquitin–proteasome was further proven in a CuONPs pulmonary exposure mice model. Together, this study uncovers a novel regulatory mechanism of NRF2 activation by protein degradation machineries in response to CuONPs exposure, which opens a novel intriguing scenario to uncover therapeutic strategies against NPs-induced vascular injury and disease.

**Graphical Abstract:**

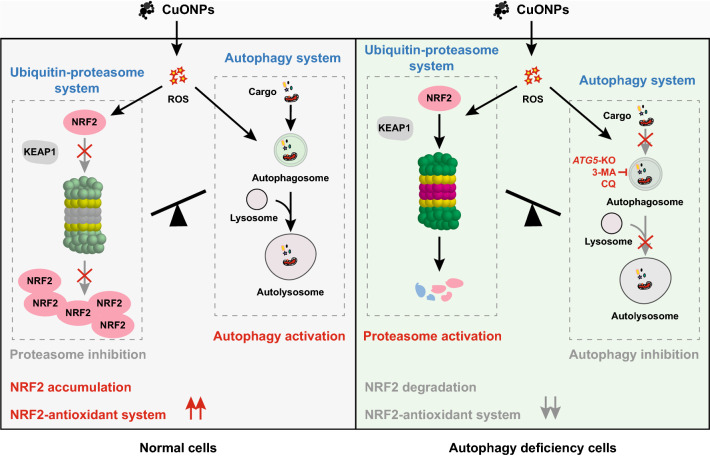

## Highlights

1. CuONPs exposure activates NRF2 signaling in vascular endothelial cells and mouse thoracic aorta.
2. KEAP1 is dispensable for NRF2 activation in CuONPs-treated vascular endothelial cells.
3. CuONPs-induced autophagy facilitates NRF2 activation in vascular endothelial cells and mouse thoracic aorta.
4. Autophagy and ubiquitin–proteasome reciprocally regulate NRF2 activation in CuONPs-treated vascular endothelial cells and mouse thoracic aorta.

## Introduction

Nanoparticles (NPs) refer to ultrafine particles less than 100 nm at least one dimension. Because of their excellent physical–chemical properties such as small size, large surface area and photothermal effect, NPs show outstanding prospects for application in industrial, medical and commercial fields [1–4]. Unfortunately, the mass production and application of NPs cause a serious threat to human health [5, 6]. Accumulating evidence has also shown that pulmonary inhaled NPs can penetrate across the pulmonary air-blood barrier and entry into circulation system, consequently induce and/or aggravate cardiovascular injury and multiple cardiovascular diseases [7–13]. Nevertheless, the interplay of cardiovascular effects and pulmonary inhaled NPs is not yet fully elucidated.

Copper oxide NPs (CuONPs) are important metal oxide NPs, which are applied as supercapacitors, sensors, solar cells, catalysis and nano-energetic materials. CuONPs are highly toxic NPs compared to many other metal oxide NPs [14]. In our previous studies, we revealed that pulmonary inhaled CuONPs triggered oxidative stress and acute lung injury in mice [15]. Pulmonary exposure to CuONPs also caused neurotoxicity because of inflammation, oxidative damage and mitochondrial dysfunctions in the cerebral cortex [16]. Meanwhile, our in vitro studies showed that CuONPs exposure damaged lysosomal functions and mitochondrial dynamics, causing excessive mitochondrial reactive oxygen species (mtROS), and triggering oxidative DNA damage and cell death in vascular endothelial cells [17–19]. However, the protection mechanisms of vascular endothelial cells in response to CuONPs exposure is still largely undefined.

The excessive accumulation of ROS, which are mainly derived from the impairment of mitochondrial oxidative respiration, are well documented as the major nanotoxicity mechanism [5, 20, 21]. NPs-induced ROS playsbidirectional roles in cell fate determination. Excessive ROS cause oxidative stress, damage plasma membrane integrity and disrupt cellular ions homeostasis, consequently triggering cell death [5, 22]. On the contrary, appropriate ROS function as beneficial signals that activate antioxidant pathway and protect against NPs-induced toxicity [20]. Nuclear factor erythroid 2-related factor 2 (NFE2L2 or NRF2) has been found playing important roles in maintaining redox homeostasis through regulating the transcription levels of components of the detoxification and antioxidant systems [23]. Considerable studies have reported that NRF2 is involved in the in vitro and in vivo toxicity of inhaled NPs [24–27]. However, the molecular mechanisms of NRF2 activation in NPs-induced vascular toxicity are not yet fully understood.

Ubiquitin–proteasome and autophagy are two major protein degradation systems in mammalian cell. The ubiquitin–proteasome is the major lysosome-independent system and is responsible for the removal of misfolded proteins [28]. Recent studies show that the ubiquitin–proteasome machinery is a crucial system mediating NRF2 activation in oxidative and electrophilic stressed cells [29]. In normal cells, NRF2 is maintained at a low protein level as it interacts with Kelch-like ECH-associated protein 1 (KEAP1), which negatively regulates NRF2 levels through promoting the degradation of NRF2 via ubiquitin–proteasome in unstressed conditions [30]. However, NRF2 is liberated from KEAP1-NRF2 complex under oxidative and electrophilic stresses, and then translocates into nucleus to transcriptionally activate downstream antioxidant genes [29]. Autophagy is another cellular degradation systems that degrades misfolded proteins and damaged organelles in the lysosome [31]. Autophagy also participates in NRF2 activation. The phosphorylation of autophagy receptor SQSTM1 (also named p62) markedly promotes the affinity binding of SQSTM1 to KEAP1, resulting in the degradation of KEAP1 in autophagy-lysosome system and then triggering the stabilization and activation of NRF2 [32]. Interestingly, there are multiple subtle and complex interplay between proteasomal and autophagic degradation systems [33]. Previous reports showed that CuONPs enhanced autophagy activity and autophagy inhibition exacerbated CuONPs-induced toxicity effects [15, 34]. In spite of this, it remains poorly understood whether the crosstalk between CuONPs-mediated autophagy and proteasome system is linked to NRF2 signaling activation in NPs-induced vascular toxicity.

Here, we investigated the roles and the regulatory mechanisms of NRF2 in vascular endothelial injury induced by inhaled CuONPs. We showed that NRF2 protected vascular endothelial cells against CuONPs-induced oxidative damage. Furthermore, we revealed that autophagy had a significant role in stabilizing NRF2 and transcriptionally activating downstream genes. Mechanistically, CuONPs-induced autophagy inhibited the ubiquitin–proteasome machinery, prevented the proteasome-dependent degradation of NRF2 and consequently resulted in the activation of NRF2 antioxidant pathway. Our study explored a novel interplay between proteasomal/autophagic degradation systems and NRF2 activation, and proposed a potential strategy against inhaled NPs-induced vascular injury.

## Material and methods

### Materials and reagents

The materials and reagents used in this study are as follows: CuONPs (Cat#544868, Sigma, St. Louis, MO, USA), MG132 (Cat#S1748, Beyotime, Shanghai, China), arsenite (Cat#H4525, Xiya Reagent Co. Ltd., Shandong, China). NAC (N-acetyl-L-cysteine, Cat#S0077, Beyotime), DHE (dihydroethidium, Cat#S0063, Beyotime), 7-AAD (7-aminoactinomycin D, Cat#AP104, MultiSciences, Hangzhou, China), MitoSOX (Cat#M36008, Thermo Fisher Scientific, Waltham, MA, USA), Calcein-AM (Cat#sc-203865, Santa Cruz Biotechnology, Santa Cruz, CA, USA), CHX (cycloheximide, Cat#AC466, Genview, Houston, TX, USA), tBHQ (tert-butylhydroquinone, Cat#HY-100489, MedChemExpress, Shanghai, China), 3-MA (3-methyladenine, Cat#HY-19312, MedChemExpress), CQ (chloroquine diphosphate salt, Cat# C6628, Sigma), Wort (wortmannin, Cat#S2758, Selleck Chemicals, Houston, TX, USA), DMEM (Dulbecco's Modified Eagle Medium, Cat#C11995500BT, Gibco, Grand Island, NY, USA), FBS (fetal bovine serum, Cat#S711-001S, Lonsera, Uruguay), penicillin–streptomycin (Cat#15140122, Thermo Fisher Scientific) and puromycin (Cat#P8230, Solarbio, Beijing, China).

### Cell culture and CuONPs exposure

Human umbilical vein endothelial cell line (HUVECs) was obtained from the American Type Culture Collection (Rockville, MD, USA). Human embryonic kidney cell line (HEK293T) was obtained from the National Collection of Authenticated Cell Cultures (Shanghai, China). All cells were cultured within the DMEM that contained the 10% FBS and penicillin–streptomycin antibiotics (100 U/ml) at 37 °C with 5% CO_2_. Stable knockout/knockdown cell lines were constructed using LentiCRISPRv2 plasmid (Cat#52961, Addgene, Watertown, MA, USA). The recombinant plasmids lentiCRISPRv2-sg*NRF2*, lentiCRISPRv2-sg*ATG5*, LentiCRISPRv2-sg*KEAP1* plasmid were constructed in our laboratory and sequenced at Sangon Biotech (Shanghai, China). Then these sequenced plasmids were co-transfected with psPAX2 (Cat#12260, Addgene) and pMD2.G (Cat#12259, Addgene) into HEK293T cells for lentiviral packaging. The HUVECs was infected with lentivirus for 48 h and then subjected to two rounds of puromycin selection to obtain stable cell lines. For CuONPs in vitro treatment, CuONPs was firstly diluted in sterilized water at dose of 2 mg/ml and ultrasonicated in a water bath for 30 min. Then, the cells seeded in 12-well plate were treated with different concentration of CuONPs for indicated time points.

### Immunoblotting

The cells were washed twice with cold PBS (phosphate buffer saline) and directly lysed with cold lysis buffer (2% sodium dodecyl sulfate, 5% β-mercaptoethanol, 0.5% sucrose and 0.2% bromophenol blue). The aortic tissues were weighed and lysed with cold RIPA lysis buffer (Cat# P0013B, Beyotime). Then, the tissue samples were homogenized using glass homogenizers and centrifuged at 14,000 × g for 15 min at 4 °C. The supernatants were collected for immunoblotting analysis. The protein lysates were separated using polyacrylamide gel electrophoresis. Separated proteins were transferred to a PVDF membranes (Merck Millipore, Billerica, MA, USA) and immunoblotted with indicated antibodies. The blotting signals were detected by BeyoECL Star kit (Cat#P0018AM, Beyotime). The following antibodies were used: NRF2 (1:1,000, Cat#16396-1-AP, Proteintech, Wuhan, China), HMOX1 (1:3,000, Cat#66743-1-Ig, Proteintech), GCLM (1:1,000, Cat#ab126704, abcam, Cambridge, MA, USA), SLC7A11 (1:1,000, Cat#ab175186, abcam), γH2AX (phospho-histone H2A.X, 1:3,000, Cat# 9718, Cell Signaling Technology, Danvers, MA, USA), KEAP1 (1:3,000, Cat#10503–2-AP, Proteintech), LC3B (1:3,000, Cat#L7543, Sigma Aldrich), SQSTM1 (1:3,000, Cat#P0067, Sigma), ATG5 (1:1,000, Cat#66744-1-Ig, Proteintech), Ubiquitin (1:3,000, Cat#D058-3, MBL, Tokyo, Japan), GAPDH (1:5,000, Cat#ab181602, abcam), β-Actin (1:10,000, Cat#HC201-01, TransGen biotech), HRP-linked anti-rabbit IgG (1:10,000, Cat#7074S, Cell Signaling Technology) and HRP-linked anti-mouse IgG (1:10,000, Cat#7076S, Cell Signaling Technology). The signal intensity of the bands was quantified using Image J software (NIH, Bethesda, MD, USA).

### Fluorescence activated cell sorting (FACS)

The cells were seeded into 12-well plate for overnight. After CuONPs treatment for indicated time, the cells were detached from culture plate by trypsin digestion and then incubated with fluorescent chemical probes diluted in PBS at room temperature for 15–30 min. DHE was used to detect cellular superoxide anions. 7-AAD was used to detect cell viability. The FACS experiments were performed in a CytoFLEX Platform (Beckman Coulter, Miami, FL, USA). All FACS results were analyzed using FlowJo™ v10 Software (BD Biosciences, San Jose, CA, USA).

### Immunohistochemistry (IHC)

Mice aorta were collected and fixed with formalin. After paraffin embedding, the tissues were and cut into 5 μm sections slides. Then, the slides were deparaffinized and rehydrated using xylene and gradient ethanol, treated with Tris/EDTA pH 9.0 buffer for heat-induced epitope retrieval. Next, the slides were treated with 3% hydrogen peroxide to quench endogenous peroxidase and blocked with 10% goat serum to reduce nonspecific binding. Followingly, section slides were incubated with MMP-2 antibody (Proteintech, Cat#10373-2-AP) and then HRP-linked anti-rabbit IgG (1:10,000, Cat#7074S, Cell Signaling Technology). After washing with tris-buffered saline tween-20 (TBST), the slides were incubated with 3,3′-diaminobenzidine (DAB) (Beyotime, Cat#P0203) and observed under a fluorescence microscope (Olympus IX53, Tokyo, Japan).

### Quantitative PCR (qPCR)

Total RNA was extracted by FastPure Cell/Tissue Total RNA Isolation Kit V2 (Cat#RC112-01, Vazyme, Nanjing, China) and reversely transcribed into cDNA using HiScript II Q RT SuperMix for qPCR (+ gDNA wiper) kit (Cat#R233-01, Vazyme). qPCR was performed using ChamQ Universal SYBR qPCR Master Mix (Cat#Q711-03, Vazyme) in a CFX Connect™ Real-Time PCR Detection System (Bio-Rad, Hercules, CA, USA). *TBP* (TATA-box binding protein) gene was used as reference gene. The data were analyzed with the 2^−ΔΔCt^ method.

### Confocal microscopy

The cells were fixed with cold 4% paraformaldehyde for 15 min and then permeabilized with 0.2% Triton X-100 for 15 min at room temperature. After blocking with BSA (bovine serum albumin) buffer (2% BSA and 0.3 M glycine in PBS) for 1 h at room temperature, the cells were incubated with primary antibodies against NRF2 (1:100, Cat#16396-1-AP, Proteintech) for overnight at 4 °C. After washing with PBS, the cells were incubated with Alexa Fluor 594 donkey anti-rabbit IgG secondary antibody (1:500, Cat#A21207, Invitrogen, Carlsbad, CA, USA) and 4',6-diamidino-2-phenylindole (DAPI, Cat#D3571, Invitrogen) for 1 h at room temperature. Finally, cells were sealed with nail polish and detected under a Nikon A1R confocal microscope (Nikon, Tokyo, Japan).

### Animal treatment

Healthy C57BL/6 J male mice (age 6–10 weeks, weight 18–20 g) were obtained from the Byrness Weil biotech Ltd (Chongqing, China). Animal experiments in this study were approved by the Institutional Animal Care and Use Committee of Chongqing Medical University. The animals were randomly divided into the following 4 groups: Control group, CuONPs group (5 mg/kg), 3-MA group (15 mg/kg) and CuONPs + 3-MA group. After 7-day adaption, the group of CuONPs animals were intratracheally instilled with a single dose of 5 mg/kg CuONPs. 3-MA group were pretreated with 3-MA via intraperitoneally administration 1 h before intratracheal instillation of CuONPs. The mice were sacrificed 3 days after CuONPs treatment and the thoracic aorta were obtained for further experiments.

### Statistical analysis

Unpaired Student’s *t*-test and one-way ANOVA followed by Tukey multiple comparison test were used for statistical analysis in this study. The data are shown as the mean ± standard deviation (S.D.). Each experiment was repeated at least three times. All statistical tests were conducted using Prism 9 software (GraphPad Software, San Diego, CA, USA). “*”, “**” and “***” represent “*P* ≤ 0.05”, “*P* ≤ 0.01” and “*P* ≤ 0.001”, respectively.

## Results

### CuONPs exposure activates NRF2 signaling pathway in vascular endothelial cells.

**Fig. 1:**
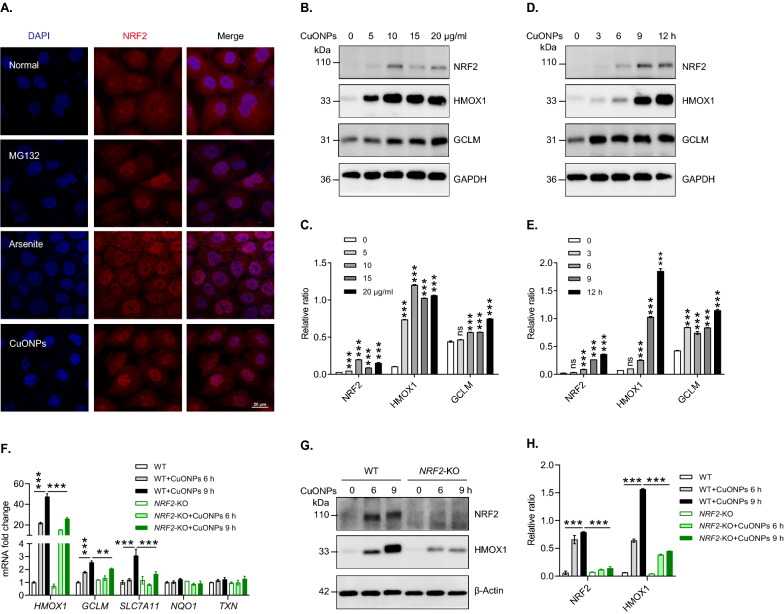
The activation of NRF2 in CuONPs-treated HUVECs. **A** Representative confocal images of NRF2 in HUVECs cells treated with CuONPs (20 μg/mL) for 12 h. Scale bar, 20 μm. Nuclei were stained with DAPI. MG132 or arsenite were used as positive controls. **B**, **C** Immunoblotting analysis and quantification of protein levels of NRF2 and its downstream HMOX1 and GCLM in HUVECs cells treated with 0, 5, 10, 15 and 20 μg/mL CuONPs for 12 h, respectively. GAPDH served as the internal control. **D**, **E** Immunoblotting analysis and quantification of protein levels of NRF2, HMOX1 and GCLM in HUVECs cells treated with 20 μg/mL CuONPs for 0, 3, 6, 9 and 12 h, respectively. GAPDH served as the internal control. **F** qPCR analysis of the mRNA levels of *HMOX1*, *GCLM*, *SLC7A11*, *NQO1* and *TXN* in wild-type (WT) or *NRF2* knockout (*NRF2*-KO) cells treated with 20 μg/mL CuONPs for 0, 6 and 9 h, respectively. **G**, **H** Immunoblotting analysis and quantification of NRF2 and HMOX1 protein levels in WT or *NRF2*-KO cells treated with 20 μg/mL CuONPs for 0, 6 and 9 h, respectively. β-Actin was used as loading control. All data are representative of three independent experiments. In **C** and **E**, Student’s *t*-test was used for statistical analysis. In **F** and **H**, one-way ANOVA followed by a Tukey multiple comparison test was used for statistical analysis. The values are expressed in mean ± S.D. *ns* not significance; **”, *P* ≤ 0.01; “***”, “*P* ≤ 0.001”

NRF2 is a crucial transcription factor that regulates cellular antioxidant response, which is recruited into nucleus under oxidative stresses and transcriptionally activates downstream genes such as *HMOX1*, *GCLM*, *SLC7A11* and *TXN* [30]. Our previous studies founded that CuONPs exposure caused ROS accumulation and oxidative damage in vascular endothelial cells [19, 35]. Here, we investigated whether NRF2 was activated under CuONPs-induced oxidative stress in HUVECs. The immunofluorescence images showed that MG132 and arsenite (two positive inducers of NRF2) and CuONPs triggered NRF2 translocation from the cytoplasm to the nucleus in HUVECs **(**Fig. 1A). Then, immunoblotting results showed that NRF2 was upregulated under CuONPs treatment and promoted the expression of HMOX1 and GCLM in a dose-dependent (Fig. 1B, C) and time-dependent manner (Fig. 1D, E). In particular, HMOX1 was the most upregulated protein compared to other proteins in CuONPs-treated cells, indicating that HMOX1 can be recognized as a indicator for NRF2 activation in the following experiments (Fig. 1D, E). Subsequently, *NRF2* knockout HUVECs cell line was constructed to reversely verify that CuONPs exposure indeed induced *NRF2* activation in HUVECs. The qPCR results showed that CuONPs treatment increased the mRNA expression levels of *HMOX1*, *SLC7A11* and *GCLM*, while the up-regulation trends were inhibited in *NRF2* knockout cell lines (Fig. 1F). Correspondingly, *NRF2* knockout also obviously prevented the upregulation of HMOX1 induced by CuONPs (Fig. 1G, H). These data indicate that CuONPs activate NRF2 signaling pathway in vascular endothelial cells.

### CuONPs-induced ROS contributes to NRF2 signaling pathway activation

**Fig. 2:**
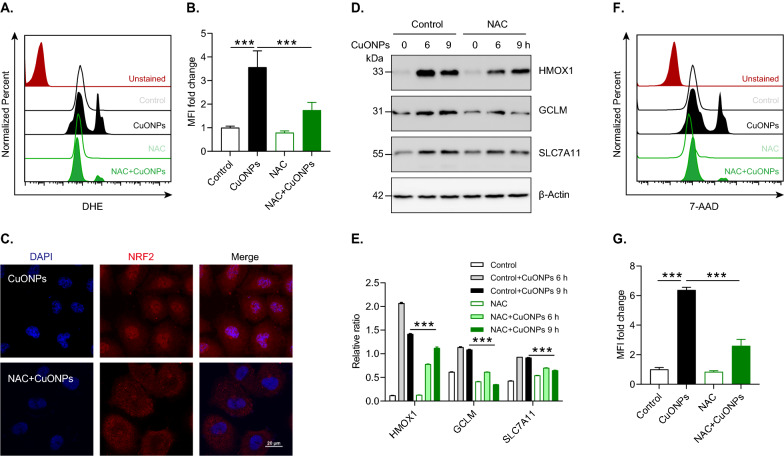
Oxidative stress induces NRF2 activation in CuONPs-treated HUVECs. **A** Representative FACS results of HUVECs cells stained with DHE. HUVECs cells were pretreated with NAC (10 mM) for 1 h and then treated with 20 μg/ml CuONPs for 12 h. Unstained, non-labeled cells. Control, normal culture media. MFI, mean fluorescence intensity. **B** Quantification analysis of DHE intensity in **A**. **C** Representative confocal images of HUVECs cells treated with NAC (10 mM) for 1 h, followed by treatment with 20 μg/mL CuONPs for 12 h. Scale bar, 20 μm. Nuclei, DAPI. **D**, **E** Immunoblotting analysis and quantification of protein levels of HMOX1, GCLM, SLC7A11 and β-Actin (loading control) in HUVECs cells treated with NAC (10 mM) for 1 h and then CuONPs (20 μg/mL) for 6 h or 9 h. **F**, **G** 7-AAD fluorescence intensity analysis of HUVECs cells treated with NAC and CuONPs for 12 h. All data are representative of three independent experiments. Statistical significance was evaluated using one-way ANOVA followed by a Tukey multiple comparison test. The values are expressed in mean ± S.D. “***”, “*P* ≤ 0.001”

To identify whether oxidative stress contributed to NRF2 signaling pathway activation in CuONPs-treated HUVECs, the cells were pretreated with NAC (a potent antioxidant) prior to CuONPs treatment. FACS results showed that ROS was significantly elevated in CuONPs-treated HUVEC cells, while NAC treatment indeed scavenged intracellular excessive ROS induced by CuONPs (Fig. 2A, B). The immunofluorescence staining results showed that NAC prevented the translocation of NRF2 from cytoplasm to the nucleus induced by CuONPs (Fig. 2C). Immunoblotting results demonstrated that the protein levels of NRF2 downstream genes in CuONPs-treated cells were downregulated by NAC, indicating CuONPs-induced ROS contributes to NRF2 signaling pathway activation (Fig. 2D, E). Finally, FACS results showed that NAC significantly alleviated CuONPs-induced cell death in HUVECs, suggesting NRF2-mediated antioxidant effects protect against CuONPs-induced cytotoxicity (Fig. 2F, G).

### NRF2 signaling protects vascular endothelial cells from CuONPs-triggered oxidative stress and cell death

**Fig. 3:**
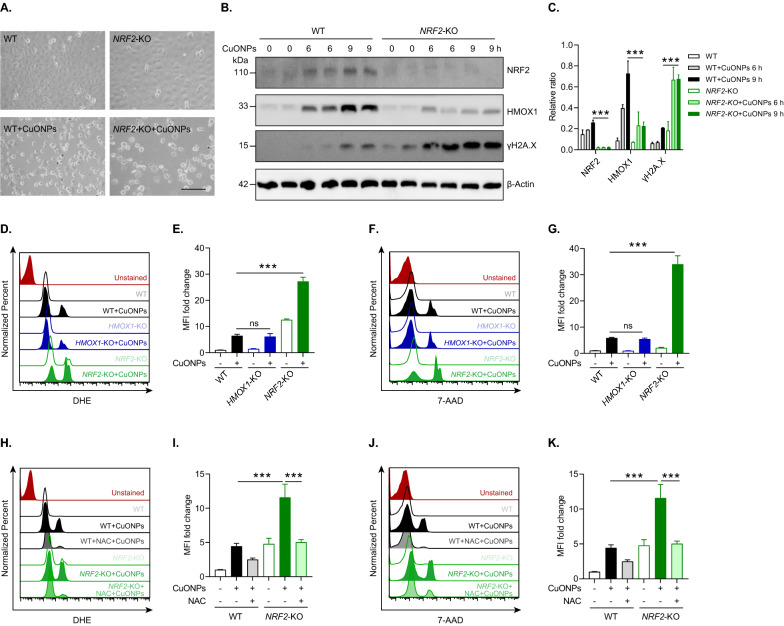
NRF2 protects against CuONPs-triggered oxidative stress and cell death in HUVECs. **A** Representative images of cell morphology of WT and *NRF2*-KO cells treated with 20 μg/ml CuONPs for 12 h. Scale bar, 100 μm. **B**, **C** Immunoblotting analysis and quantification of protein levels of NRF2, HMOX1, γH2AX and β-Actin (loading control) in WT and *NRF2*-KO cells treated with 20 μg/mL CuONPs for 0, 6 and 9 h, respectively. **D**, **E** FACS analysis and quantification of DHE intensity in WT, *HMOX1* knockout (*HMOX1*-KO) or *NRF2*-KO cells treated with CuONPs (20 μg/ml) for 12 h, respectively. Unstained, non-labeled cells. MFI, mean fluorescence intensity. **E**, **F** FACS analysis and quantification of 7-AAD intensity in WT, *HMOX1*-KO and *NRF2*-KO cells treated with CuONPs (20 μg/ml) for 12 h, respectively. **H**, **I** FACS analysis and quantification of DHE intensity in WT and *NRF2*-KO cells treated with NAC (10 mM) for 1 h before treatment with CuONPs (20 μg/ml) for 12 h, respectively. **J**, **K** FACS analysis and quantification of 7-AAD intensity in WT and *NRF2*-KO cells treated with NAC and CuONPs. All data are representative of three independent experiments. Statistical significance was evaluated using one-way ANOVA followed by a Tukey multiple comparison test. The values are expressed in mean ± S.D. *ns* not significance; “***”, “*P* ≤ 0.001”

Previous studies have revealed that NRF2 plays a critical protective role in response to oxidative stress-induced cell death such as apoptosis, ferroptosis and necrosis [36–39]. Here, we investigated whether NRF2 protected vascular endothelial cells against CuONPs-induced cytotoxicity. Cell morphology images showed that HUVECs became round and detached from the culture plate indicating cell toxicity induced by CuONPs. Moreover, *NRF2* knockout (*NRF2*-KO) obviously aggravated CuONPs-induced cells rounding-up and detachment, suggesting that NRF2 played a crucial role in mitigating CuONPs-induced cytotoxicity (Fig. 3A). The phosphorylation of H2A.X at ser 139 (γH2AX) is a marker of DNA double-strand break (DSB) [40]. Immunoblotting results showed that CuONPs increased the protein level of γH2AX in a time-dependent manner in HUVECs cells, and caused more γH2AX accumulation in *NRF2*-KO HUVECs cell line (Fig. 3B, C). FACS results showed that *NRF2* knockout increased the fluorescent intensity of DHE, suggesting more superoxide anions accumulation in CuONPs-treated *NRF2*-KO cells (Fig. 3D, E). Then, the increases of fluorescent intensity of dead cell probe 7-AAD indicated that *NRF2* knockout aggravated CuONPs-induced vascular endothelial cells death (Fig. 3F, G). Intriguingly, the knockout of *HMOX1* (a major downstream gene of NRF2) did not increase the levels of cellular superoxide anions and cell death in CuONPs-treated cells, compensation mechanisms existent when HMOX1 knockout to maintain redox balance. Next, we found that antioxidant NAC significantly decreased the levels of cellular superoxide anions in CuONPs-treated *NRF2*-KO cells (Fig. 3H, I), and considerably reduced CuONPs-induced cells death in *NRF2*-KO HUVECs cell line (Fig. 3J, K). Collectively, these results suggest that NRF2 regulates ROS homeostasis and protects vascular endothelial cells against CuONPs-induced oxidative injury and cell death.

### KEAP1 is not primarily involved in NRF2 activation in CuONPs-treated vascular endothelial cells

**Fig. 4:**
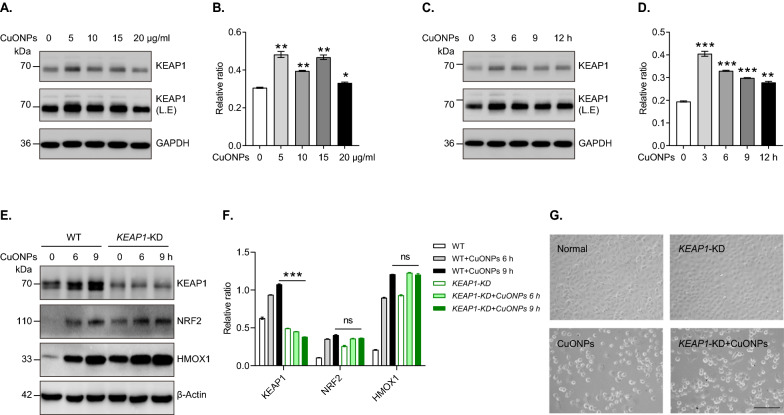
The roles of KEAP1 in CuONPs-induced NRF2 activation. **A**, **B** Immunoblotting analysis and quantification of KEAP1 protein levels in HUVECs cells treated with 0, 5, 10, 15, 20 μg/mL CuONPs for 12 h, respectively. L.E, longer exposure time. GAPDH served as an internal control. **C**, **D** Immunoblotting analysis and quantification of KEAP1 protein levels in HUVECs cells treated with 20 μg/mL CuONPs for 0, 3, 6, 9 and 12 h, respectively. **E**, **F** Immunoblotting analysis and quantification protein levels of KEAP1, NRF2, HMOX1 and β-Actin (loading control) in WT and *KEAP1* knockdown (*KEAP1*-KD) cells treated with 20 μg/mL CuONPs for 0, 6 and 9 h, respectively. ns, not significance. **G** Representative images of cell morphology of WT and *KEAP1*-KD cells treated with 20 μg/ml CuONPs for 12 h. Scale bar, 100 μm. In **B** and **D**, Student’s *t*-test was used for statistical significance. In **F**, statistical significance was evaluated using one-way ANOVA followed by a Tukey multiple comparison test. All data are representative of three independent experiments. The values are expressed in mean ± S.D. *ns* not significance; “*”, *P* ≤ 0.05; **”, *P* ≤ 0.01; “***”, “*P* ≤ 0.001”

As a substrate receptor for cullin3-dependent E3 ubiquitin ligase, KEAP1 is a negative regulator of NRF2 [30]. Here, we detected the protein levels of KEAP1 in CuONPs-treated HUVECs. Intriguingly, the immunoblotting results showed that the KEAP1 failed to be degraded in ubiquitin–proteasome system, but slightly increased after CuONPs treatment (Fig. 4A–D). To investigate whether KEAP1 was involved in the activation of NRF2 in CuONPs-treated HUVECs cells, *KEAP1* knockdown (*KEAP1*-KD) cell lines were constructed based on CRISPR/Cas9 gene editing system. We found that *KEAP1* knockdown moderately increased NRF2 protein level in CuONPs-untreated cells (unstressed conditions), but it did not significantly further upregulate NRF2 and HMOX1 after CuONPs treatment for 9 h (Fig. 4E, F). Cell morphology observation showed that there was no obvious difference of cell viability between CuONPs-treated HUVECs and CuONPs-treated *KEAP1*-KD HUVECs (Fig. 4G). Overall, these data reveal KEAP1 is not primarily involved in NRF2 activation in CuONPs-treated vascular endothelial cells.

### Autophagy is involved in CuONPs-induced NRF2 signaling pathway activation

**Fig. 5:**
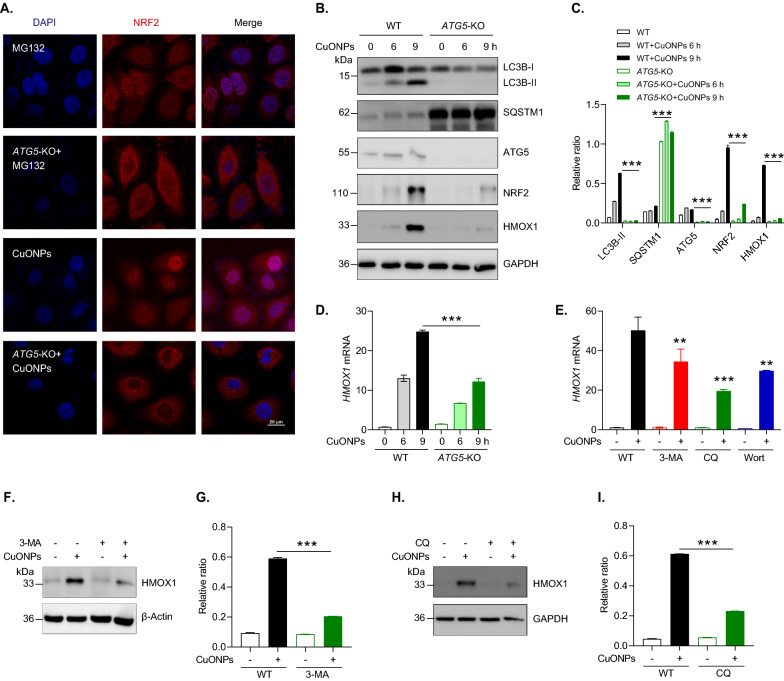
Autophagy is involved in CuONPs-induced NRF2 activation. **A** Representative confocal images of NRF2 subcellular location in WT and *ATG5* knockout (*ATG5*-KO) cells treated with CuONPs (20 μg/mL) or MG132 (20 μM) for 12 h, respectively. Scale bar, 20 μm. Nuclei, DAPI. **B**, **C** Immunoblotting analysis and quantification of protein levels of NRF2, HMOX1, ATG5, SQSTM1, LC3B and GAPDH (loading control) in WT and *ATG5*-KO cells treated with 20 μg/mL CuONPs for 0, 6 and 9 h. **D** qPCR analysis of *HMOX1* mRNA levels in WT or *ATG5*-KO cells treated with 20 μg/mL CuONPs for 0, 6 and 9 h. **E** qPCR analysis of *HMOX1* mRNA levels in HUVECs cells treated with CuONPs (20 μg/mL) with or without 3-MA (5 mM), CQ (10 μM) and Wort (2.5 μM), respectively. **F**, **G** Immunoblotting analysis and quantification of HMOX1 protein levels in HUVECs cells pretreated with 3-MA (5 mM) for 1 h and treated with CuONPs (20 μg/mL) for 12 h. β-Actin was used as internal control. **H**, **I** Immunoblotting analysis and quantification of HMOX1 protein levels in HUVECs cells pretreated with CQ (10 μM) for 1 h and treated with CuONPs (20 μg/mL) for 12 h. GAPDH was used as internal control. All data were analyzed using one-way ANOVA followed by a Tukey multiple comparison test. All data are representative of three independent experiments. The values are expressed in mean ± S.D. **”, *P* ≤ 0.01; “***”, “*P* ≤ 0.001”

Several lines of evidence indicate that autophagy is triggered by ROS signaling, which feedback-regulates NRF2-mediated antioxidative response [41]. Here, we determined whether autophagy was involved in NRF2 activation in CuONPs-treated HUVECs. Immunofluorescence staining showed that *ATG5* knockout (*ATG5*-KO) obviously prevented NRF2 nucleus translocation induced by MG132 and CuONPs in HUVECs (Fig. 5A). Immunoblotting results showed that the protein levels of NRF2 and its downstream HMOX1 were downregulated in CuONPs-treated *ATG5*-KO HUVECs compared with CuONPs-treated WT HUVECs cells (Fig. 5B, C). qPCR results further confirmed that *ATG5* knockout considerably inhibited the transcriptional upregulation of *HMOX1* induced by CuONPs (Fig. 5D). Furthermore, we verified the interplay between autophagy and NRF2 activation using several autophagy chemical inhibitors such 3-methyladenine (3-MA), wortmannin (Wort) and chloroquine (CQ). qPCR results showed that all selected autophagy inhibitors significantly repressed the transcriptional upregulation of *HMOX1* induced by CuONPs in HUVECs (Fig. 5E). Immunoblotting results confirmed that 3-MA and CQ indeed repressed CuONPs-induced HMOX1 upregulation (Fig. 5F–I). Taken together, we demonstrate that autophagy participates in NRF2 signaling activation in CuONPs-treated HUVECs.

### Autophagy and ubiquitin–proteasome reciprocally regulate NRF2 in CuONPs-treated cells

**Fig. 6:**
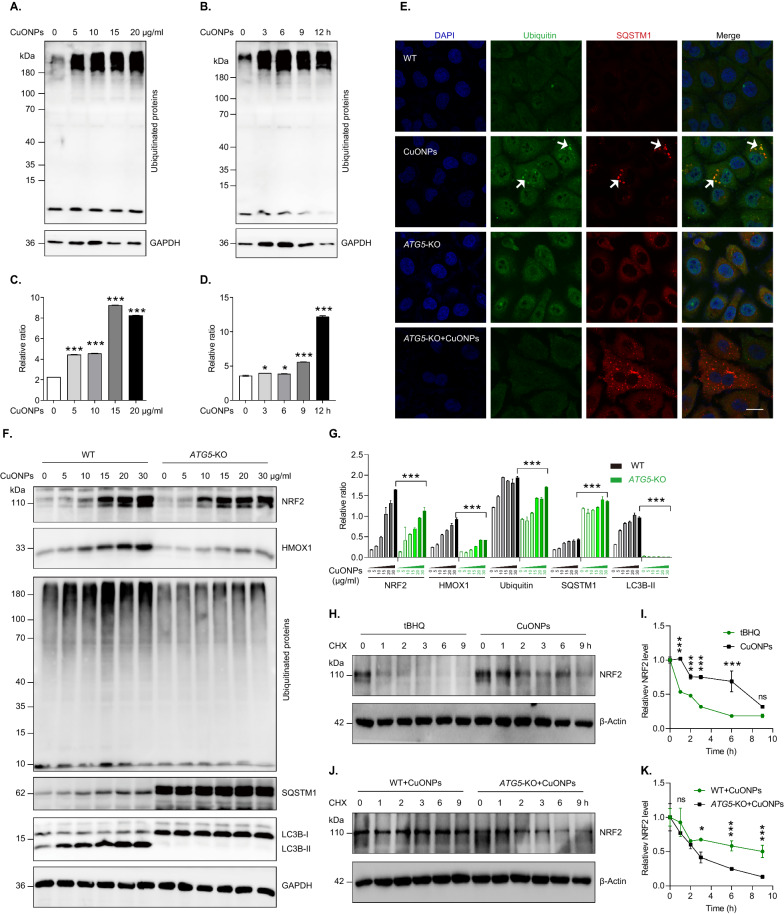
Autophagy inhibition activates ubiquitin–proteasome pathway in CuONPs-treated cells. **A** and **C** Immunoblotting analysis and quantification of ubiquitinated proteins levels in HUVECs treated with 0, 5, 10, 15 and 20 μg/ml CuONPs for 12 h. GAPDH was used as loading control. **B** and **D** Immunoblotting analysis and quantification of ubiquitinated proteins levels in HUVECs treated with CuONPs (20 μg/ml) for 0, 3, 6, 9 and 12 h, respectively. GAPDH was used as loading control. **E** Representative confocal images of WT and *ATG5*-KO cells treated with CuONPs (20 μg/ml), respectively. The cells were immunofluorescently stained and analyzed with ubiquitin and SQSTM1 antibody. **F** and **G** Immunoblotting analysis and quantification of the levels of NRF2, HMOX1, ubiquitinated proteins, SQSTM1, LC3B and GAPDH (loading control) in WT and *ATG5*-KO cells treated with 0, 5, 10, 15, 20 and 30 μg/ml CuONPs for 12 h, respectively. **H** and **I** Immunoblotting analysis and quantification of NRF2 half-life in CuONPs-treated HUVECs. Cells were treated with tBHQ (10 μM) and CuONPs (20 μg/ml) for 9 h, and then treated with CHX (50 μg/ml) for 0, 1, 2, 3, 6, 9 h, respectively. β-Actin served as loading control. **H** Immunoblotting analysis and quantification of NRF2 half-life in CuONPs-treated WT or *ATG5*-KO HUVECs cells. Cells were treated with tBHQ (10 μM) and CuONPs (20 μg/ml) for 9 h, and then treated with CHX (50 μg/ml) for 0, 1, 2, 3, 6, 9 h, respectively. β-Actin served as loading control. In **C** and **D**, Student’s *t*-test was used for statistical significance. In **G**, **I** and **K**, statistical significance was evaluated using one-way ANOVA followed by a Tukey multiple comparison test. All data are representative of three independent experiments. The values are expressed in mean ± S.D. *ns* not significance; “*”, *P* ≤ 0.05; “***”, “*P* ≤ 0.001”

Accumulating evidence suggests that both autophagy and ubiquitin–proteasome are involved in NRF2 activation [33], thus we investigate whether there is a crosstalk between autophagy and proteasome in regulating NRF2 activation in CuONPs-treated vascular endothelial cells. Firstly, we showed that CuONPs exposure inhibited ubiquitin–proteasome system characterized by the significant increase of ubiquitinated proteins in CuONPs-treated HUVECs (Fig. 6A–D). Then, immunofluorescence staining showed that protein aggregates (marked by ubiquitin and SQSTM1 antibodies) obviously accumulated in CuONPs-treated HUVECs cells, but these aggregates were cleared in CuONPs-treated *ATG5*-KO cells (Fig. 6E). Furthermore, we revealed that *ATG5* knockout remarkably accelerated the degradation of ubiquitinated proteins and simultaneously decreased NRF2 levels in HUVECs induced by CuONPs, suggesting autophagy promoted NRF2 activation probably via inhibiting ubiquitin–proteasome system (Fig. 6F, G). Next, we investigated NRF2 half-life in CuONPs-treated cells through cycloheximide chase assay. tBHQ (a NRF2 activator) was used a positive control for NRF2 induction. Cycloheximide (an inhibitor of protein synthesis) was used to monitor NRF2 degradation rate in CuONPs-treated cells. Immunoblotting results showed that the protein half-time of NRF2 was less than 2 h in tBHQ-treated cells but over 6 h in CuONPs-treated cells, suggesting CuONPs exposure prevented NRF2 degradation in HUVECs (Fig. 6H, I). Furthermore, cycloheximide chase assay showed that NRF2 degraded faster in CuONPs-treated *ATG5*-KO HUVECs cell line (Fig. 6J, K). Taken together, these data reveal that autophagy plays a crucial role in NRF2 stability via inhibiting proteasome-dependent degradation of NRF2 in CuONPs-treated cells.

### Autophagy inhibition promotes proteasome-dependent Nrf2 degradation in CuONPs pulmonary exposure mice model

**Fig. 7:**
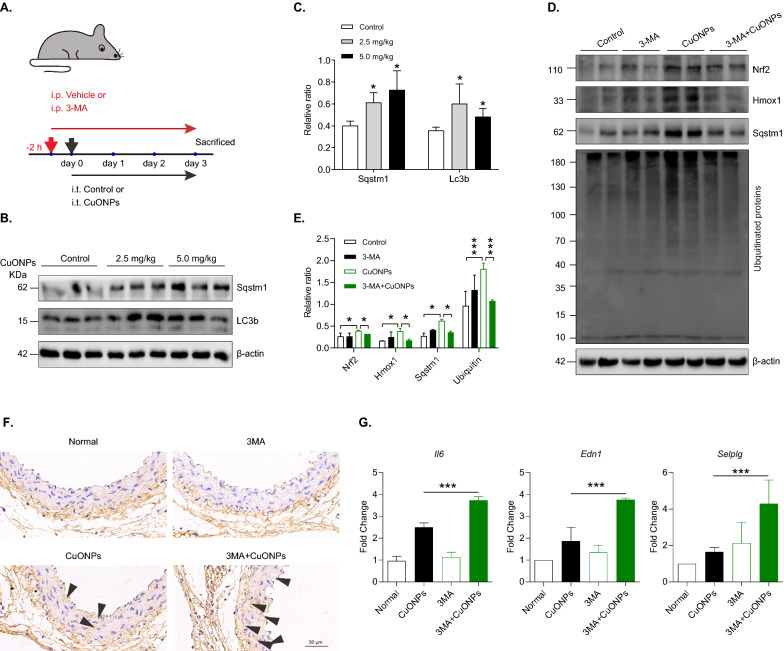
Autophagy inhibitor accelerates proteasome-dependent degradation of Nrf2 in mice. **A** Schematics of the in vivo experimental workflow. C57BL/6 J mice were treated with vehicle (PBS) or PBS diluted 3-MA (15 mg/kg) for 2 h via intraperitoneal injection (i.p.), and then exposed to CuONPs via intratracheal instillation (i.t.) for 3 days. **B**, **C** Mice were instilled intratracheally with CuONPs for 3 days. Immunoblotting analysis and quantification of protein levels of Sqstm1, LC3b and β-actin (loading control) in mice aorta tissues. **D**, **E** Mice were pretreated intraperitoneally with or without 3-MA and then instilled intratracheally with CuONPs for 3 days. Immunoblotting analysis and quantification of protein levels of Ubiquitin, Nrf2, Hmox1, Sqstm1 and β-actin (loading control) in mice aorta tissues. **F** Representative images of immunohistochemistry using antibodies against MMP-2 in the intima and media region of abdominal aorta. Black arrows indicate high expression regions of MMP-2. Scale bar, 50 μm. **G** The mRNA expression levels of *Il6*, *Edn1* and *Selplg* in mouse aorta. In **C**, Student’s *t*-test was used for statistical significance. In **E** and **G**, one-way ANOVA followed by a Tukey multiple comparison test was used for statistical significance. All data are representative of three independent experiments. The values are expressed in mean ± S.D. “*”, *P* ≤ 0.05; “***”, “*P* ≤ 0.001”

We then investigated the role and related mechanisms of Nrf2 (NRF2 homolog in mice) activation in a pulmonary CuONPs-exposed mice model. As shown in Fig. 7A, C57BL/6J mice were instilled intratracheally with low (2.5 mg/kg) or high (5 mg/kg) dose of CuONPs as described by our previous study [15]. For 3-MA inhibition, mice were injected intraperitoneally with vehicle (PBS) control or 3-MA (15 mg/kg) 2 h before CuONPs pulmonary exposure. After 3 days exposure, mice were sacrificed and the thoracic aorta were collected for following experiments. Immunoblotting results showed that CuONPs pulmonary exposure obviously increased the protein levels of autophagy marker Lc3b and Sqstm1 in aorta tissues, suggesting autophagy was involved in CuONPs-induced vascular injury (Fig. 7B, C). However, autophagy inhibitor 3-MA obviously promoted the degradation of ubiquitinated proteins and accelerated the proteasome-dependent degradation of Nrf2 in pulmonary CuONPs-treated mice (Fig. 7D, E). Then, we showed that inhaled CuONPs induced vascular inflammation characterized by the upregulation of matrix metalloproteinase 2 (MMP-2) in vascular intima and media, and the inhibition autophagy through small molecule inhibitor 3-methyladenine (3MA) exacerbated CuONPs-induced inflammatory response (Fig. 7F). In parallel, the transcription levels of inflammatory factors were obviously upregulated in 3MA plus CuONPs-treated mice compared with CuONPs control mice, including *Il-6*, *Edn1* and *Selplg* (Fig. 7G). These data indicate autophagy inhibition promotes proteasome-dependent Nrf2 degradation and may aggravate CuONPs pulmonary exposure-induced mice vascular injury.

## Discussion

NRF2 is a transcription factor that transcriptionally regulates the expression of antioxidant proteins, detoxifying enzymes and anti-inflammatory factors in response to environmental pollutants or pathological changes [42, 43]. In this study, we investigated the roles and the underlying mechanisms of NRF2 activation in vascular injury induced by inhaled NPs. We confirmed that NRF2 signaling was an important antioxidant system against CuONPs-induced vascular injury. In vitro CuONPs exposure remarkably upregulated NRF2 signaling and its downstream antioxidant targets in vascular endothelial cells (Fig. 1). Moreover, we revealed that intracellular ROS induced by CuONPs contributed to NRF2 activation (Fig. 2). We also revealed that inhaled CuONPs promoted NRF2 activation in mice thoracic aorta (Fig. 7D, E). In addition, we illustrated that NRF2 knockout aggravated CuONPs-induced oxidative stress, DNA damage and cell death in vascular endothelial cells (Fig. 3). This finding is consistent with previous studies showing that NRF2 is activated in response to NPs exposure and NRF2 deletion sensitizes cells or mice to NPs-induced inflammation and oxidative injury [25, 26, 44, 45]. It is well documented that NRF2 is essential for maintaining physiological functions in the cardiovascular system and NRF2 deficiency is linked to multiple vascular diseases such as atherosclerosis, hypertension and diabetes. NRF2 deficiency reduces fibrous cap thickness and promotes features of atherosclerotic plaque instability [46]. Angiotensin II, a risk factor for hypertension, can activate NRF2 singling and NRF2 activator alleviates vascular dysfunction in hypertension [47]. NRF2 also participates in diabetic wound healing as evidenced by the delayed wound closure rates in *NRF2* knockout mice but the significant improvement of diabetic wound healing after pharmacological activation of the NRF2 [48]. These data reveal that NRF2 is a potential sensor protein for predicting vascular injury caused by multiple risk factors including inhaled NPs and highlight that NRF2 may be a candidate therapeutic target for alleviating vascular related diseases induced by NPs exposure.

KEAP1-NRF2 axis is a canonical pathway for NRF2 activation. KEAP1 is a negative regulator of NRF2, which interacts with NRF2 and recruits it to ubiquitin–proteasome for degradation in unstressed cells. However, oxidative and electrophilic stress disrupt the KEAP1-NRF2 complex conformation and accelerate KEAP1 proteasome-dependent degradation, resulting in NRF2 nucleus translocation for transcriptionally regulating antioxidant genes expression [29, 49]. It has been shown that KEAP1 deletion increases NRF2-medicated antioxidant gene expression, and then attenuates smoking-induced oxidative stress and lung damage [50]. Meanwhile, deletion of KEAP1 in primary human T lymphocytes promotes Treg cell activation and can potentially be used for treating immune-related diseases [51] KEAP1 deletion also upregulates NRF2 downstream protein SLC7A11, causing the increase of NADPH consumption and glucose dependency in lung cancer cells [52]. Whether KEAP1 participates in NPs exposure-triggered NRF2 activation is largely undefined. Yin et al. reported that KEAP1 was significantly decreased in human epidermal keratinocyte line HaCaT in response to zinc oxide NPs (ZnONPs) exposure [53]. Weng et al. demonstrated that ceria NPs (CNPs) restored the redox homeostasis via decreasing KEAP1 expression and then negatively regulating NRF2 protein level in acute kidney injury [54]. Unexpectedly, we recently founded that in vitro exposure to ZnONPs caused the accumulation of both NRF2 and KEAP1 in HUVECs [45]. In the current study, we further showed that KEAP1 also failed to be degraded but slightly increased in CuONPs-induced oxidative conditions in vascular endothelial cells (Fig. 4A–D). We speculate the difference of KEAP1 expression pattern in response to NPs may be mainly attributed to the difference of cells and mice model. Hereafter, we constructed a *KEAP1* knockout cell line based on CRIPSR/Cas9 system to investigate whether KEAP1 was involved in NRF2 activation. Unfortunately, we failed to obtain a homozygous *KEAP1* knockout HUVECs cell line after two rounds of puromycin selection. One plausible explanation was *KEAP1* knockout might lead to lethality to vascular endothelial cells. Thus, we selected the heterozygous knockout cells (*KEAP1* knockdown) for following experiments. Indeed, *KEAP1* knockdown did not significantly affect NRF2 signaling and downstream targets (Fig. 4E, F). Furthermore, we showed that *KEAP1* knockdown failed to rescue CuONPs-triggered HUVECs cells death (Fig. 4G). These data suggest KEAP1 is not primarily involved in NRF2 antioxidant pathway activation in NPs-induced vascular injury.

Autophagy is a highly coordinated process which plays a crucial pro-survival role under conditions of nutrient starvation and extracellular stress. Previous studies have illustrated that autophagy is a noncanonical mechanism of NRF2 activation [55, 56]. NRF2 and its downstream genes were upregulated in autophagy-deficient mice. Mechanistically, autophagy deficiency resulted in the remarkable accumulation of autophagy receptor SQSTM1 and the upregulation the phosphorylation of SQSTM1 on serine 351 (corresponding to serine 349 in mice). Phosphorylated SQSTM1 competes with NRF2 to bind with KEAP1 and recruit it degradation in autophagy pathway[32]. Growing evidence have highlighted NPs as a novel class of autophagy activators [57]. It has been demonstrated that ZnONPs, silica NPs (SiNPs) and silver NPs (AgNPs) trigger a functional autophagy response which feedback-regulates cell fates in NPs-treated cells. [58–60]. Autophagy is also activated by CuONPs and functioned as a protective signal against CuONPs-induced lung epithelial cells [61]. Our recent in vivo and in vitro studies revealed that autophagy deficiency exacerbated CuONPs-induced acute lung injury and significantly aggravated CuONPs-induced vascular endothelial cells death [15, 18]. Hence, it is very necessary to investigate whether CuONPs-induced autophagy participates in NRF2 activation. Intriguingly, we showed that inhibition of autophagy by *ATG5* knockout inhibited NRF2 nuclear translocation in CuONPs-treated HUVECs (Fig. 5A). Moreover, we found that *ATG5* knockout indeed significantly upregulated SQSTM1, but unexpectedly decreased NRF2 level and inhibited the transcription of NRF2 downstream gene *HMOX1* (Fig. 5B–D). Consistently, several autophagy inhibitors 3-MA, Wort and CQ prevented *HMOX1* transcription in CuONPs-treated HUVECs (Fig. 5E–I). Our data are consistent to a recent report uncovering that arsenic activated NRF2 pathway in primary human prostate epithelial cells via an autophagy-dependent manner [62]. These data indicate autophagy is involved in CuONPs-mediated NRF2 activation in HUVECs via a novel singling pathway but not classical SQSTM1-KEAP1-NRF2 pathway.

In this study, we showed that the protein levels of NRF2 and KEAP1 in HUVECs were both upregulated by CuONPs exposure (Figs. 1, 4). However, no significant differences of the mRNA level of *NRF2* and *KEAP1* were founded between control and CuONPs-treated HUVECs (data not shown). These results suggest NRF2 activation mainly derived from the inhibition of NRF2 degradation in CuONPs-treated cells. It is now well-accepted that autophagy and ubiquitin–proteasome are two major cellular quality control system responsible for degradation of misfolded proteins [63]. A recent report suggests that AgNPs exposure caused protein thiol oxidation and aggregation in mesenchymal triple-negative breast cancer cell line SUM159 [64]. Moreover, embryonic exposure to AgNPs significantly altered the expression of multiple genes involved in ubiquitin–proteasome machinery [44]. Sara Nahle et al. found that proteasome subunits such as PSMA2, PSMD8 and PSMD5 in macrophages were upregulated in response to carbon nanotubes exposure [65]. Our recent study showed that inhaled ZnONPs induced the accumulation of ubiquitinated proteins in the aortic endothelium of mouse [45]. In the present study, we further revealed that CuONPs exposure significantly increased ubiquitinated proteins in HUVECs (Fig. 6A–D). We then investigated the NRF2 half-life in CuONPs-treated cells through a cycloheximide chase assay. The results obviously showed that NRF2 degradation was inhibited by CuONPs exposure, indicating ubiquitin–proteasome pathway was disturbed after CuONPs exposure (Fig. 6H–I). Notably, autophagy and ubiquitin–proteasome systems are functionally interconnected in eukaryotic cells [33]. Previous findings showed that the impairment of ubiquitin–proteasome machinery activated autophagy and the KEAP1-NRF2 pathway [66]. Correspondingly, Wang et al. reported that autophagy inhibition remarkably increased proteasomal activities and upregulated the protein levels of several proteasomal subunits such as PSMB5 [67]. Recently, Kim et al. presented evidence that deubiquitinating enzyme USP14 functioned as a significant determinator between autophagy and ubiquitin–proteasome, because USP14 inhibition enhanced proteasome degradation activity but negatively feedback blocked autophagic flux [68]. Here, we determined whether autophagy was involved in the degradation of NRF2 in CuONPs-treated HUVECs. We showed that autophagy deficiency through knocking out *ATG5*, coding an essential component of the autophagy machinery, remarkably accelerated the clearance of ubiquitinated proteins in CuONPs-treated HUVECs (Fig. 6F, G). We founded that NRF2 degradation was obviously accelerated in *ATG5* knockout cells and autophagy inhibitor-treated mice (Figs. 6J, K and 7D, E). More importantly, we revealed that inhibition autophagy through small molecule inhibitor 3MA exacerbated CuONPs-induced inflammatory response in mouse aorta (Fig. 7F, G). These data suggest CuONPs-mediated autophagy is a crucial signaling for NRF2 activation via negative-feedback inhibition of proteasome-dependent NRF2 degradation. And, autophagy-mediated NRF2 activation protected against CuONPs-induced vascular inflammatory injury.

Despite this, the molecular mechanism underlying the compensatory negative-feedback of autophagy and ubiquitin–proteasome are not fully elucidated in this study. We look forward to address this concern in our ongoing study.

## Conclusion

In the current study, we found that NRF2 was activated in vascular endothelial cells after CuONPs pulmonary exposure. Oxidative stress which derived from CuONPs exposure participated in NRF2 signaling activation, while NRF2 knockout significant aggravated NPs-induced oxidative stress and cell death in vascular endothelial cells. Mechanistically, we revealed that autophagy participated in NRF2 activation, because autophagy inhibition accelerated degradation of NRF2 in ubiquitin–proteasome system. Our study uncovers a novel reciprocal links between autophagy and ubiquitin–proteasome system in regulating NRF2 activation after CuONPs exposure, and suggests their important implications for preventing inhaled NPs-triggered vascular injury.

## Data Availability

The data used to support the findings of this study are available from the corresponding author upon request.
